# More flexible and more innovative: the impact of flexible work arrangements on the innovation behavior of knowledge employees

**DOI:** 10.3389/fpsyg.2023.1053242

**Published:** 2023-04-26

**Authors:** Liqun Jiang, Zhiyuan Pan, Yunshi Luo, Ziyan Guo, Deqiang Kou

**Affiliations:** International Business School, Jinan University, Zhuhai, Guangdong, China

**Keywords:** flexible work arrangements, innovation behavior, thriving at work, knowledge employees, HRM-opportunity

## Abstract

Flexible work arrangements (FWA) are becoming increasingly widespread as an efficient means of coping with a dynamic and competitive business environment. Existing studies have primarily examined the impact of FWA as a management system; however, its impact on employee innovation behavior has not been fully explored. Based on the self-determination theory, this study constructed a moderated mediation model that empirically examined the influence of FWA on the innovation behavior of knowledge employees. Our findings are as follows: (1) FWA can activate innovation behavior among knowledge employees; (2) thriving at work plays a partial mediating role; (3) human resource policies that facilitate opportunities have a positive moderating effect. The findings fill a theoretical research gap and provide insights for managers on implementing FWA to promote the innovative behavior of knowledge employees.

## 1. Introduction

Flexible work arrangements (FWA), generally being referred to flexibility of spatial (where work is conducted) and/or temporal (when work is conducted) (Rau and Hyland, 2002), have become prevalent in industrialized nations, particularly Europe and the U.S. For instance, according to the U.S. Department of Labor, by the end of the 20th century, approximately 27% of full-time U.S. employees had flexible working hours to some extent, and approximately 50% of employees were able to telecommute. This situation expanded dramatically following the COVID-19 outbreak. To minimize the negative impact of epidemic prevention and control measures on organizational operations, many companies in developing countries implemented FWA. Such measures included allowing employees to work from home or adjust their office hours. According to an experiment in Ctrip, a Chinese company, working from home increased employee performance by 13% as well as increased job satisfaction and led to a 50% drop in turnover rate (Bloom et al., 2015). Concurrently, the pandemic has promoted the use of digital work tools and online office systems, breaking the temporal and spatial constraints of traditional work patterns. Thus, the technical tools required for FWA to be effective are becoming increasingly mature and ubiquitous. FWA is becoming a trend by which organization respond to the dynamic and competitive environment and mobilize employees’ motivation.

Research in developed countries have examined FWA-related topics, such as its antecedents (Swanberg et al., 2005) and influencing mechanisms and impacts (Lambert et al., 2008). They explored FWA from three perspectives. First, the meaning of different types of FWA (e.g., flexibility with regard to work time, work location, or job content) is conducive to building a deeper understanding of its theoretical implications as a management system (Beers, 2000; Thompson et al., 2015). The second approach looks at FWA from the employee’s perspective, examining aspects such as demographic characteristics as antecedents of FWA and behavioral performance outcomes (Halpern, 2005; Swanberg et al., 2005). Most studies from this perspective have found that employees have positive attitudes toward FWA and show positive outcomes. For example, Ziderman (2020) showed that FWA positively impacts employees’ job satisfaction and motivation. In contrast, Chung and van der Lippe (2020) found that FWA is conducive to balancing family and work relationships. The third research approach takes the organizational perspective, examining organizational antecedents (e.g., organizational culture characteristics) (Lyness et al., 2012; Masuda et al., 2012), and organizational outcomes (e.g., performance or competitiveness) (Stavrou, 2005; Wahab and Tatoglu, 2020). However, the results of these studies indicate that FWA as a work arrangement design at the organizational level is complex and difficult to analyze. For example, Ter Hoeven and Zoonen (2015) argued that FWA brings both advantages and challenges to employee well-being and organizational performance (Rubery et al., 2016). Existing studies have mainly examined the meaning and impact of FWA as a workplace management system. A growing number of scholars have gradually emphasized the role of FWA in motivating employees and enhancing organizational competitiveness (Peretz et al., 2018). Though, studies have shown that a relaxed and autonomous work environment can motivate employees to invest more resources and capabilities in innovation (Wallace et al., 2016), existing research does not fully explain how FWA activates employees’ innovative behaviors, especially among knowledge employees, who are the primary drivers of innovation. Thus, it remains unclear whether the temporal and spatial flexibility of FWA encourage knowledge employees to engage in innovative work more proactively. Accordingly, we aim to by investigate whether FWA has a motivational influence on knowledge employees’ innovation behaviors.

The theoretical base of this study is provided by the self-determination theory, which suggests that an individual’s environment can enhance their autonomy and competence, which can, in turn, enhance and stimulate their intrinsic motivation and facilitate the internalization of extrinsic motivation. Accordingly, this study suggests that FWA plays a role in stimulating employees’ intrinsic motivation through two mechanisms. First, FWA provides employees with more job autonomy in work practices, as shown by Lott (2020). Second, it greatly enhances employees’ feelings of autonomy, given their spatial and temporal work flexibility, and reduces restrictions (Mache et al., 2020). These two mechanisms strengthen employees’ behavioral self-determination, build their intrinsic potential for self-development and self-fulfillment, and stimulate their intrinsic motivation. This study suggests that such a process also motivates knowledge employees to innovate independently.

This study also examined the role of thriving at work as part of this process. According to Spreitzer et al. (2005), the characteristics of a work situation and related work resources jointly stimulate employees’ thriving at work. Li et al. (2022) found that thriving at work benefits individuals’ physical and mental health and prevents job burnout. Furthermore, thriving at work is positively related to employees’ innovation behavior (Wallace et al., 2016). When employees exhibit proactive work behaviors, their work resources will, in turn, promote thriving at work (Wrzesniewski and Dutton, 2001). There is a strong link between employees’ thriving at work and innovative behavior. Thus, thriving at work mediates between FWA and employee innovation behavior.

Furthermore, organizational climate or conditions often influence innovation behavior, especially innovation-related human resource management (HRM) policies and practices. Appelbaum et al. (2000) proposed the ability, motivation, and opportunity (AMO) model of HRM, which suggests that employees’ proactive work performance is closely related to three dimensions of HRM policies and practices: the ability facilitation dimension (HRM ability), the motivation facilitation dimension (HRM motivation), and the opportunity facilitation dimension (HRM opportunity). These three dimensions are complementary; however, each impacts the outcome variable differently. In contrast to the other two dimensions, policies and practices under the HRM-opportunity dimension focus on creating the necessary conditions for knowledge-intensive teamwork by building required competencies and motivations (Chuang et al., 2016). Such policies are needed to support knowledge employees’ innovation behavior. Previous research has demonstrated a substantial relationship between HRM-opportunity and employee innovation behavior (Ozbag et al., 2013). Therefore, we chose the HRM opportunity dimension as a moderating variable in the theoretical model.

Based on the discussion above, this study makes two significant theoretical contributions. First, our study reveals that FWA is conducive to stimulating the innovative behavior of knowledge employees, which extends the research perspective of the consequences of FWA to some work behavior with certain job characteristics. Existing studies have focused on the impact of FWA on broad work behaviors common to all employees, such as absenteeism, turnover, and work satisfaction, or work-related attainment, such as work-family balance. These studies did not distinguish between the work characteristics of different jobs. Although some studies have suggested that FWA can stimulate employees’ proactive work behaviors (Mache et al., 2020), no study has tested whether it affects innovative behavior. We propose that FWA is associated with the innovative behavior of knowledge employees, extending Mache et al. (2020) study on idiosyncratic jobs that the flexibility of work content by individualized work contracts (i.e., job duties that fit the individual’s abilities or interests) would bring out adaptive innovation value for the organization. We suggest that FWA be specifically applied to target groups with specific job characteristics, thereby enhancing their innovative performance. Our study fills the theoretical gap by extending the research perspective and further affirms the positive impact of FWA. Second, the current study reveals the internal mechanism by which FWA stimulates knowledge employees’ innovation behavior, using thriving at work as a mediating variable. This provides new theoretical insights by responding to Chen and Fulmer (2018) call for further research on the mediating mechanism of FWA.

The findings of this study have several important practical implications. First, it contradicts the negative perception of FWA (common in developing countries) as a means by which organizations can extend working hours without limitations. The actual application of FWA can improve employee satisfaction, as previous studies have revealed, and benefit their innovative behavior, as our study showed. Both of them will have a significantly positive effect on organizational performance in the long run. Furthermore, our study suggests that organizations can practice FWA in a planned manner. Job characteristics are the most important considerations when activating employees’ innovation potential. HRM policies should be implemented to support FWA.

## 2. Theory and hypotheses

### 2.1. Impact of FWA on knowledge employees’ innovation behavior

Continued adherence by companies to a fixed work system makes it more difficult for their employees to autonomously arrange their work time, especially for the knowledge employees who have difficulty working owing to their physiological condition or who have specific needs related to personal affairs or family life (Piszczek and Pimputkar, 2021). Mental tension is also caused by the fact that in a formal work environment, personnel is always under the supervision of managers. It is difficult for individuals who are physically and mentally fatigued or in a bad frame of mind to take the initiative and become more innovative. In contrast, FWA creates a good working environment and reduces work conflict and stress (Yunus and Mostafa, 2021), which encourages employees to proactively invest useful resources such as time, attention, and energy into their work. Grzywacz et al. (2008) showed that FWA significantly improved employee wellness, which in turn helped improve their work engagement.

Compared to other employees, knowledge employees may have a stronger desire to realize their self-worth and enjoy challenging work. They prefer a pleasant, autonomous work atmosphere to stressful conditions (Spivack and Milosevic, 2018). FWA significantly promotes their perception of a relaxed and inclusive organizational environment and reduces excessive and wasteful consumption of their psychological resources. Some researchers argue that a relaxed and enjoyable work environment enhances employee innovation performance (Shalley et al., 2004; Bailey et al., 2017; Siyal et al., 2021). FWA provides employees greater discretion over their work schedules and office locations, which boosts their job autonomy. They have the authority to make their own decisions and allocate their resources. Hence, the more empowered an organization’s employees are, the stronger the positive influence of job autonomy on employee innovation (Spivack and Woodside, 2019; Gao et al., 2020). Additionally, they have a stronger feeling of responsibility and commitment and are eager to assume more tasks and obligations (Brammer et al., 2007).

Owing to their experience of job autonomy from FWA, knowledge employees have greater job autonomy and will to boost their innovation performance. We propose the following hypothesis:

*H1*: *FWA* is positively related to knowledge employees’ *innovation behavior*.

### 2.2. The mediating role of thriving at work

Grzywacz et al. (2008) and Hill et al. (2008) found that perceived work flexibility is negatively related to stress and burnout, and lower stress enables employees to have healthier physical and mental states (Bayighomog et al., 2021). Thus, FWA strengthens a strong sense of thriving at work by reducing employee stress. Spreitzer and Porath’s (2013) model clearly indicates that thriving at work has two dimensions, learning and vitality, which are correlated to innovation. When employees feel a sense of learning, it leads to their positive development. Enrichment and progress in their professional knowledge increase their confidence and creativity, encouraging them to go beyond the status quo and apply new things into work. Likewise, when employees feel vitality, they have more energy and motivation to implement new ideas and may try to achieve professional breakthroughs.

Especially for knowledge employees, FWA gives them more autonomy to coordinate their jobs with other affairs, which helps them to have more opportunities to learn and communicate. Additionally, they can easily maintain high vitality levels when they feel more control over their working hours or places. So, they maintain a good situation of thriving at work and have more innovative behaviors, ultimately contributing to the organization’s innovative development. Accordingly, we propose the following hypothesis:

*H2*: *Thriving at work* mediates the relationship between *FWA* and knowledge employees’ *innovation behavior.*

### 2.3. The moderating effect of HRM-opportunity

Knowledge employees’ innovation behavior is built on a continuous search for inspiration and innovation opportunities. HRM policies and practices that facilitate this kind of opportunity include job rotation, information sharing, employee engagement, teamwork, and strengthening employees’ social networks inside and outside organizations. HRM opportunity creates more opportunities for knowledge flows and information exchange, facilitating innovation among knowledge employees. For example, job rotation can expose employees to new work environments and job content; information sharing can enable employees to gain new knowledge; employee engagement and teamwork promote team innovation and collaboration; and external interactions may contain new knowledge to inspire innovation. Therefore, under a high HRM opportunity scenario, knowledge employees who thrive in the workplace may discover more possibilities for innovation. Additionally, opportunity-facilitating HRM methods help create and maintain emotional bonds within the organization by fostering connections and communication among employees, making it easier for employees to obtain support and assistance from colleagues (Chuang et al., 2016).

Thus, it can be expected that high HRM opportunity not only increases the individual incentive of employees with FWA to innovate but also increases team support for their innovations. Consequently, we propose the following hypothesis:

*H3*: *HRM opportunity* moderates the indirect effect between *FWA* and knowledge employees’ *innovation behavior* through *thriving at work*.

## 3. Materials and methods

### 3.1. Survey

This study examined knowledge employees’ engagement in producing, creating, diffusing, and applying knowledge and bringing value-added intellectual capital to organizations. Considering the above literature review, the sample for this study includes knowledge employees who hold positions in R&D, design, engineering, quality, production management, marketing, procurement, finance, and human resources within organizations. Since we targeted knowledge employees, non-organization personnel (e.g., freelancers) and non-knowledge employees (e.g., front-line production workers) were excluded. We cooperated with organizations that have applied FWA even before the pandemic and are strongly interested in research, mainly from the manufacturing and IT industries, based on the following considerations: the application and development of FWA in developing countries such as China is still in its infancy, and is mainly concentrated in the manufacturing and IT industries. Furthermore, innovation is valued and common in these industries. Considering the common application of digital working platforms after the pandemic, we distributed electronic versions of the questionnaire in Chinese with the help of these organizations’ human resource managers through their internal working systems. A letter attached before the questionnaire explained that the university conducted the investigation for research goals, and each participant had the right to refuse to give a response.

A small-scale pilot study with a sample of 100 knowledge employees was conducted to further check and refine the measures. The investigation period lasted from October 2021 to December 2021, with 500 questionnaires distributed and 488 returned. The questionnaires were screened as follows: first, questionnaires with incomplete or incorrect answers (e.g., ticking two answers to a single-choice question) were excluded; second, questionnaires with a strong regularity of answers (e.g., answers 3-3-3-3-3 or 1-2-3-4-5) were excluded; again, the answers to the reverse test questions were checked and excluded without consistent answers, and if the length of time to fill in the questionnaire was too short (for not thinking carefully about the implications of the questions) or too long (for the possible interruption in filling the electronic questionnaires), the questionnaire was regarded as unreliable and rejected. A total of 429 valid questionnaires were obtained, representing a response rate of 85.8%.

As shown in Table 1, the gender distribution of the respondents is close to 1:1, and their ages are mainly concentrated in the age groups 26–30 and 31–35, in line with the average age of employees engaged in the IT and manufacturing industries (Morrison and Murphy-Hill, 2013). Regarding education level, 73.4% of the respondents had a bachelor’s degree, which represents the average education level among knowledge employees. The organizations in which the respondents worked were mainly private or state-owned, accounting for 66.4 and 25.6%, respectively. In terms of job types, R&D/design employees accounted for the highest percentage (41.0%), which is useful for this study which primarily targeted innovation behaviors among R&D/design employees. Regarding family characteristics, 71.3% of the respondents said they had dependent minor children, which is consistent with the age distribution of the sample. The sample was distributed evenly across all categories without extreme cases for the remaining characteristics. In conclusion, the sample met the requirements of the study design.

**Table 1:** Basic information of the samples (*N* = 429).

Variables	Category	Frequency	Percentage (%)	Variables	Category	Frequency	Percentage (%)
Gender	Female	204	47.60	Company size	Less than 100 people	35	8.20
	Male	225	52.40		100 ~ 300 people	147	34.30
Age	25 years old and below	31	7.20		300 ~ 1,000 people	170	39.60
	26 ~ 30 years old	147	34.30		More than 1,000 people	77	17.90
	31 ~ 35 years old	168	39.20	Type of work	R&D/Design	176	41.00
	36 ~ 40 years old	41	9.60		Production/Engineering/Quality	114	26.60
	41 ~ 45 years old	16	3.70		Sales/Marketing	55	12.80
	46 years old and above	26	6.10		Other civilian jobs	84	19.60
Education level	University Specialists	60	14.00	Management Level	Non-management positions	116	27.00
	Undergraduate	315	73.40		Grassroots Management	147	34.30
	Master and above	54	12.60		Middle Management	131	30.50
Nature of business	State-owned company	110	25.60		Senior Management	35	8.20
	Private company	285	66.40	Length of employment in this company	Less than 1 year	19	4.40
	Foreign-owned company	34	7.90		1 ~ 3 years	65	15.20
Have any minor children	There are	306	71.30		3 ~ 5 years	127	29.60
	None	123	28.70		5 ~ 10 years	162	37.80
					More than 10 years	56	13.10

### 3.2. Measures

We chose the established measures that are commonly adopted in existing studies. Measures of employee innovation, thriving at work, and HRM opportunity were originally developed in English and have Chinese versions. All items of the measures were scored on a 5-point Likert scale (from 1 = strongly disagree to 5 = strongly agree).

#### 3.2.1. Flexible work arrangements

Referring to Rau and Hyland (2002), Greenberg and Landry (2011), and Bloom et al. (2015), we adopted a four-item scale with a two-dimensional structure of FWA, including work time and workplace flexibility, which was originally developed by Hornung et al. (2008) and adapted to the Chinese context by Chinese studies (Liu and Wang, 2022). It includes four questions: ‘My company has a shortened workweek’, ‘The company’s employees can flexibly arrange their working hours according to their actual situation’, ‘Employees can work from home or remotely’, and ‘The company allows employees to work shifts, change shifts, or cover shifts according to specific situations’. The Cronbach’s α coefficient for this scale was 0.842.

#### 3.2.2. Employee innovation

We used a six-item scale to measure employee innovation; an example item is, ‘I am an innovative person at work’ (Scott and Bruce, 1994). The Cronbach’s α coefficient for this scale was 0.740.

#### 3.2.3. Thriving at work

We used the 10-item scale that Porath et al. (2012) developed to measure thriving at work, including two reverse-scored questions. An example item is, ‘At work, I learn a lot’. The Cronbach’s α coefficient for this scale was 0.791.

#### 3.2.4. HRM-opportunity

We used a six-item scale to measure the HRM opportunity (Chuang et al., 2016). An example item is ‘The company uses job rotation for knowledge workers to gain experience by moving them across different functional areas or divisions’. The Cronbach’s α coefficient for this scale was 0.803.

#### 3.2.5. Control variables

Referring to Wallace et al. (2016) and Wang et al. (2019), we controlled for employees’ gender, age, education level, nature of the company, company size, job type, managerial rank, length of employment in the current company, and the presence of minor children. Existing studies suggest that these variables may be correlated with employee innovation behavior.

### 3.3. Data analysis

#### 3.3.1. Descriptive statistics and correlations

Table 2 shows the means and standard deviations of each variable and the correlation coefficients between them. There were significant positive correlations between the independent variable of FWA and the mediating variable of thriving at work (*r* = 0.440, *p* < 0.01), between the mediating variable of thriving at work and the outcome variable of employee innovation (*r* = 0.626, *p* < 0.01), and between the independent variable of FWA and the outcome variable of innovation behavior (*r* = 0.494, *p* < 0.01), which provided initial support for the proposed hypothesis and enabled us to proceed with the regression analysis and moderated mediation test.

**Table 2:** Descriptive statistics and correlation analysis.

	Average value	Standard deviation	1	2	3	4	5	6	7	8	9
1. Age	2.86	1.19	1								
2. Company size	2.67	0.86	0.119^*^	1							
3. Management level	2.20	0.93	0.321^**^	0.058	1						
4. Length of employment in the company	3.40	1.04	0.609^**^	0.133^**^	0.435^**^	1					
5. Have children or not	0.71	0.45	0.315^**^	0.077	0.307^**^	0.474^**^	1				
6. FWA	3.71	0.91	0.115^*^	0.053	0.233^**^	0.289^**^	0.260^**^	1			
7. Thriving at work	4.17	0.45	0.236^**^	0.062	0.240^**^	0.365^**^	0.383^**^	0.440^**^	1		
8. Employee Innovation	4.23	0.53	0.163^**^	0.003	0.288^**^	0.344^**^	0.368^**^	0.494^**^	0.626^**^	1	
9. HRM-Opportunity	4.15	0.71	0.115^*^	0.041	0.210^**^	0.233^**^	0.368^**^	0.527^**^	0.625^**^	0.677^**^	1

^*^Denotes *p* < 0.05, ^**^denotes *p* < 0.01.

The variance inflation factor (VIF) values of each variable in the model were less than three, far lower than the critical value of 10, which is far lower than the critical value of 10, indicating that there were no serious multicollinearity issues. Thus, regression analysis is a feasible approach for testing the hypotheses.

#### 3.3.2. Common method bias analysis

Regarding the common method variance, Harman’s single one-way analysis of factors was applied. Four factors (more than one) were analyzed using Harman’s one-way test, and the explanatory power of factor 1 was only 36.949% (less than the cutoff of 50%) (Podsakoff et al., 2003), indicating that the common method deviation did not have a serious impact on our research.

#### 3.3.3. Confirmatory factor analysis

We conducted a series of confirmatory factor analyses using AMOS 26.0. A validated factor analysis was conducted on four variables to confirm the discriminant validity of the variables: FWA, employee innovation, thriving at work, and HRM opportunity. The closer the RMSEA and SRMR are to 0, the better the model fit (Marsh et al., 2004). The closer the CFI, TLI, and IFI are to 1, the better the model fit (Hu and Bentler, 1999). As shown in Table 3, the four-factor model fits the data significantly better than the other models (CFI = 0.902, TLI = 0.900, IFI = 0.902, RMSEA = 0.062, SRMR = 0.0674, and *χ*^2^/df = 2.647). Therefore, the four-factor model was the most appropriate for this study.

**Table 3:** Results of confirmatory analysis.

	*x* ^2^	df	*x*^2^ /df	RMSEA	SRMR	IFI	CFI	TLI
One factor (FWA + TH + IN+HRMO)	1537.667	299	5.143	0.098	0.0775	0.749	0.748	0.726
Two factors (FWA + TH + IN, HRMO)	1438.945	298	4.829	0.095	0.0751	0.769	0.769	0.747
Three factors (FWA, TH + IN, HRMO)	1075.005	296	3.632	0.078	0.0667	0.842	0.841	0.826
Four factors (FWA, TH, IN, HRMO)	775.621	293	2.647	0.062	0.0574	0.902	0.902	0.900

FWA, Flexible work arrangements; TH, thriving at work; IN, employee innovation; HRM, HRM-Opportunity.

## 4. Results

We used SPSS 25.0 for the following statistical analysis and PROCESS macro (Model = 4 for Hypothesis 2 and Model = 14 for Hypothesis 3) (Hayes, 2018a), which can simultaneously test complex moderation, mediation, and moderated mediation models; it generates a bootstrap confidence interval (CI) to estimate the significance of indirect effects (Hayes, 2018a).

Hypothesis 1 predicts that FWA is positively related to knowledge employees’ innovation behavior. In Table 4, the regression analysis was first performed on the outcome variable (innovation behavior) by adding control variables to the model to obtain Model 1. The independent variable FWA was then added to obtain Model 2, to test the main effect between FWA and innovation behavior. The results showed that FWA positively affects knowledge employees’ innovation behavior (*b* = 0.390, *p* < 0.001); thus, Hypothesis 1 is supported.

**Table 4:** Results of main effects.

Predictive variables	Employee Innovation	
	Model 1	Model 2
Control variables		
Age	−0.095	−0.05
Company size	−0.043	−0.05
Management level	0.146^**^	0.099^*^
Length of employment in this company	0.226^***^	0.134^*^
Have any minor children	0.249^***^	0.192^***^
Independent variable		
Flexible work schedule		0.390^***^
*R* ^2^	0.196	0.33
*F*	20.621^***^	34.591^***^

^*^Denotes *p* < 0.05; ^**^denotes *p* < 0.01; ^***^denotes *p* < 0.001.

Hypothesis 2 predicted that thriving at work mediates the relationship between FWA and knowledge employees’ innovation behavior. For robustness, we used stepwise regression and bootstrapping analyses to test the proposed mediation effect. In Table 5, we first tested the relationship between FWA and thriving at work. The results of Model 3 showed that FWA positively influenced thriving at work among knowledge employees (*b* = 0.336, *p* < 0.001). Model 4 examined the relationship between thriving at work and employee innovation behavior. The regression analysis showed that, after adding the mediating variable, there was still a significant positive relationship between FWA and innovation among knowledge employees; the absolute value of the coefficient became smaller (*b* = 0.236, *p* < 0.001). The mediating variable also has a significant positive relationship with knowledge employees’ innovation (*b* = 0.457, *p* < 0.001). Thus, the result supports the positive relationship between thriving at work and employee innovation behavior.

**Table 5:** Result of mediating effects tests.

Predictive variables	Employee innovation	Thriving at work	Employee innovation
	Model 1	Model 3	Model 4
Control variables			
Age	−0.095	0.043	−0.069
Company size	−0.043	0.004	−0.052
Management level	0.146^**^	0.026	0.087^*^
Length of employment in this company	0.226^***^	0.130^*^	0.075
Have any minor children	0.249^***^	0.212^***^	0.095^*^
Independent variable			
Flexible work schedule		0.336^***^	0.236^***^
Intermediate variables			
Thriving at work			0.457^***^
*R* ^2^	0.196	0.293	0.477
*F*	20.621^***^	29.171^***^	54.926^***^

^*^Denotes *p* < 0.05, ^**^denotes *p* < 0.01, ^***^denotes *p* < 0.001.

To ensure robustness, we further tested the mediating role of thriving at work in the relationship between FWA and employee innovation behavior. A bootstrap test was conducted using the PROCESS plug-in (Model = 4) developed by Hayes and Scharkow (2013), with a random sample of 5,000 times and a confidence interval of 95%. As shown in Table 6, the indirect effect of thriving at work reached significance at a 95% confidence interval (*b* = 0.09, CI = [0.06, 0.13]) and did not contain zero (Hayes, 2018b), indicating that the mediating effect of thriving at work was significant and hypothesis 2 was supported.

**Table 6:** Bootstrap test results for mediation effects.

Thriving at work	Effect	BootSE	95% confidence interval	
			Lower limit	Upper limit
Direct effect	0.14	0.02	0.09	0.18
Indirect effects	0.09	0.02	0.06	0.13

Hypothesis 3 predicted that HRM opportunity would moderate the indirect effect between FWA and knowledge employees’ innovation through thriving at work. As shown in Table 7, first, based on Model 1, we introduced thriving at work and HRM-opportunity to obtain Model 5. Then, we centered the mediating variable thriving at work and the moderating variable HRM-opportunity on the interaction term, respectively, and put thriving at work, HRM opportunity, and the interaction term into Model 6. The results showed that thriving at work (*b* = 0.362, *p* < 0.001), HRM-opportunity (*b* = 0.542, *p* < 0.001), interaction term (*b* = 0.228, *p* < 0.01), and knowledge employees’ innovation all had significant positive relationships with each other. Thus, HRM-opportunity positively moderates the relationship between thriving at work and innovation among knowledge employees.

**Table 7:** Results of the test for moderating effects.

Predictive variables	Employee innovation		
	Model 1	Model 5	Model 6
Control variables			
Age	−0.095	−0.066	−0.079
Company size	−0.043	−0.05	−0.054
Management level	0.146^**^	0.083^*^	0.083^*^
Length of employment in this company	0.226^***^	0.132^**^	0.127^**^
Have any minor children	0.249^***^	0.03	0.025
Independent variable			
Thriving at work		0.284^***^	0.362^***^
HEM-opportunity		0.450^***^	0.542^***^
Interaction terms			
Thriving at work × HRM-Opportunity			0.228^***^
*R* ^2^	0.196	0.552	0.582
*F*	20.621^***^	74.065^***^	73.055^***^

^*^Denotes *p* < 0.05, ^**^denotes *p* < 0.01, ^***^denotes *p* < 0.001.

**Figure 1:**
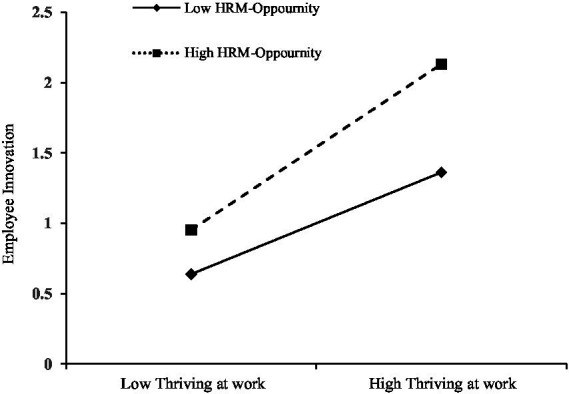
Moderating effect of HRM-opportunity.

As shown in Figure 1, the slope is greater under a high rather than a low level of HRM opportunity. Thus, compared to the condition of low HRM opportunity, the innovation behavior of employees with a high sense of thriving at work is much higher than that of employees with low thriving at work under the condition of high HRM opportunity. This finding supports the idea that HRM opportunity positively moderates the positive relationship between thriving at work and employee innovation.

The moderating variable was then tested for its moderating effect on the mediating effect of thriving at work. The bootstrap test under different HRM opportunity conditions was conducted using the PROCESS macro (Model = 14), with 95% confidence intervals and 5,000 samples. In addition, based on the judgment index method (Hayes, 2018a), the index of moderated mediation was 0.03 with a 95% CI [0.008, 0.07], which does not contain zero. Thus, it supports Hypothesis 3 is supported, which means that HRM opportunity moderates the indirect influence of FWA on the innovation behavior of knowledge employees via the mediating effect of thriving at work.

## 5. Discussion

The ongoing improvement in digital and telecommuting tools and systems has meant that the technical conditions for the practice of FWA have matured. An increasing number of businesses are providing more autonomy for employees through FWA to respond to the dynamic and competitive business environment (Jarrahi et al., 2021). Existing studies have focused on the positive effects of FWA on employees’ work behavior or performance in general (Shalley et al., 2004; Bailey et al., 2017; Siyal et al., 2021). However, few studies have examined whether FWA is conducive to motivating knowledge employees to commit more resources and energy to innovation. We constructed a moderated mediation model and empirically tested its theoretical propositions using questionnaire data to address this gap. The following conclusions were drawn:

First, FWA stimulates innovation behavior among knowledge employees through intrinsic motivating mechanisms. FWA gives knowledge employees more autonomy in work practices and enhances their feelings of autonomy (Jarrahi et al., 2021). This encourages them, giving them more motivation to seek self-fulfillment through work (Ziderman, 2020). This also increases their willingness to innovate. These findings suggest that FWA is not just a management system that can be used to improve employees’ work satisfaction; it can also activate employee innovation behavior (Riaz et al., 2019).

Second, thriving at work partially mediates between FWA and knowledge employees’ innovation behavior. Some studies have shown that FWA can relieve pressure and improve employees’ physical and mental states (Jiang et al., 2020). We extend these findings and support that with strong experiences of autonomy at work, knowledge employees have more energy and motivation to put new ideas into practice, ultimately promoting innovation.

Third, HRM opportunity positively moderates the mediating role of thriving at work. Previous research has shown that promoting opportunity-oriented HRM policies provides more innovation opportunities for knowledge employees and makes it easier for employees to obtain innovation support and trust from colleagues (Jo et al., 2020; Salas-Vallina et al., 2021). Thus, with the support of organizational resources and the recognition of colleagues, employees who maintain a sense of thriving at work are more likely to engage in innovative activities.

### 5.1. Theoretical contributions

The primary theoretical contributions of this study are as follows. Firstly, our study reveals that FWA is conducive to stimulating the innovative behavior of knowledge employees, which extends the research perspective of the consequences of FWA to some work behavior with certain job characteristics. Existing studies have focused on the impact of FWA on broad work behaviors common to all employees, such as absenteeism, turnover, and work satisfaction, or work-related attainment, such as work-family balance. These studies did not distinguish between the work characteristics of different jobs. Although some studies have suggested that FWA can stimulate employees’ proactive work behaviors (Mache et al., 2020), no study has tested whether it affects innovative behavior. We propose that FWA is associated with the innovative behavior of knowledge employees, extending Mache et al. (2020) study on idiosyncratic jobs that the flexibility of work content by individualized work contracts (i.e., job duties that fit the individual’s abilities or interests) would bring out adaptive innovation value for the organization. We suggest that FWA be specifically applied to target groups with specific job characteristics, thereby enhancing their innovative performance. It provides a new research perspective and further affirms the positive impact of FWA.

The findings also enrich research on the antecedents of employee innovation behavior. This field has focused more on specific antecedent factors (Wang et al., 2019; Albort-Morant et al., 2020), such as organizational support and personal creativity, without considering the role of the wide application of FWA. Relevant studies show that when employees feel leaders and organizations give them autonomy and support, they become more involved in their work, generate more new ideas, and improve their creativity (Giorgi et al., 2020; Slåtten et al., 2020). FWA allows employees to arrange their working hours and workplaces, which is good for balancing work pressure and personal life, which can, in turn, promote employees to implement new ideas in practice and is conducive to transforming creativity into innovation (Chung and van der Lippe, 2020; Dousin et al., 2021). Thus, our study reveals that FWA is an important antecedent of employee innovation behavior and lays a foundation for future related research.

Second, this study explored the underlying mechanism and theoretical boundary conditions between FWA and innovation behavior. Previous studies have provided plentiful discussions on the antecedents and outcomes of FWA but have not examined the internal mechanisms by which FWA plays a role in specific conditions. Chen and Fulmer (2018) argued that the apparent lack of research on the mediating mechanism by which FWA has a positive influence means that the current understanding of the impact of FWA on organizations and employees is limited. This study responds to this argument and uses thriving at work as a mediating variable and HRM opportunity as a moderating variable to explain the impact of FWA on employee innovation, providing new theoretical insights into FWA.

### 5.2. Practical implications

This study’s findings have two practical implications. First, the results may reduce public misunderstanding of FWA to some extent. It is wise for organizations to use FWA to facilitate new forms of work and ease psychological pressure on employees, which may have a range of positive effects and even mitigate labor conflicts, especially during difficult times, such as the COVID-19 pandemic. In industrialized nations, mostly in Europe and the U.S., FWA is a standard organizational management practice. In contrast, there is still a severe lack of awareness and comprehension in developing countries, especially China, where overtime work culture prevails. Leaving aside the complex causes of this variation, Chinese organizations are generally unenthusiastic about FWA and are thus missing valuable opportunities. This study shows that FWA, which allows employees to arrange their own working time or space to a certain extent, has a practical and positive effect on organizational development.

Second, the results have implications for the optimal application of FWA. In many developing countries, small- and medium-sized businesses are frequently unable to innovate owing to insufficient capital and a dearth of innovative personnel. This study proposes that organizations consider the innovation potential of different management systems and pertinent HRM policies. The use of HRM to promote innovation opportunities can intensify the role of FWA in promoting employee innovation.

### 5.3. Limitations and directions for future research

Despite these contributions, this study had some limitations. First, it is based on cross-sectional survey data and thus lacks a longitudinal perspective. This research design can only reflect correlations between variables at one point in time; the intensity of the effect of FWA on thriving at work and employee innovation may change over time. Second, our results cannot necessarily be generalized to knowledge employees from other industries owing to their different job characteristic. The subjects in this study are mainly from the manufacturing and IT industries that knowledge employees need more work autonomy for improving their innovation performance to support the organizational innovative development. This job characteristic is different from the same work positions in other industries, e.g., the service industry.

These limitations suggest future avenues for research that focus on the following aspects. First, the research could provide a richer consideration of the degree of flexibility and dynamic impact of FWA in different scenarios. For example, what degree of flexibility is necessary for enhancing innovation performance? Does FWA positively or negatively impact team (rather than individual) innovation performance? Second, future research should consider other job characteristics such as employee from the platform-based internet companies. We can extend the Bloom et al.’s (2015) research and explore how the different work flexibility (e.g., work time, work location, or job content) impacts the platform-based internet employees’ innovative behavior for they always face intensively technological or industrial change.

## Data availability statement

The original contributions presented in the study are included in the article/supplementary material, further inquiries can be directed to the corresponding author.

## Ethics statement

Ethical review and approval was not required for the study on human participants in accordance with the local legislation and institutional requirements. Written informed consent from the patients/participants or patients/participants legal guardian/next of kin was not required to participate in this study in accordance with the national legislation and the institutional requirements.

## Author contributions

LJ guided the research design and reviewed the manuscript. DK participated in the study design and data collection. LJ and DK prepared the manuscript. ZP, YL, and ZG designed the study, performed the statistical analyses, and revised the manuscript. All authors have contributed to the manuscript and approved the submitted version.

## Funding

This study was supported by the National Social Science Foundation of China (20BGL146).

## Conflict of interest

The authors declare that the research was conducted in the absence of any commercial or financial relationships that could be construed as a potential conflict of interest.

## Publisher’s note

All claims expressed in this article are solely those of the authors and do not necessarily represent those of their affiliated organizations, or those of the publisher, the editors and the reviewers. Any product that may be evaluated in this article, or claim that may be made by its manufacturer, is not guaranteed or endorsed by the publisher.
